# CNF-Functionalization as Versatile Tool for Tuning Activity in Cellulose-Derived Product Hydrogenation

**DOI:** 10.3390/molecules24020316

**Published:** 2019-01-16

**Authors:** Andrea Jouve, Stefano Cattaneo, Sofia Capelli, Marta Stucchi, Claudio Evangelisti, Alberto Villa, Laura Prati

**Affiliations:** 1 Dipartimento di Chimica, Università degli Studi di Milano, via Golgi 19, I-20133 Milano, Italy; andrea.jouve@unimi.it (A.J.); stefano.cattaneo2@unimi.it (S.C.); sofia.capelli@unimi.it (S.C.); marta.stucchi@unimi.it (M.S.); alberto.villa@unimi.it (A.V.); 2 National Council of the Research, CNR-ISTM, Via G. Fantoli 16/15, 20138 Milan, Italy; claudio.evangelisti@istm.cnr.it

**Keywords:** ruthenium, carbon, nanofiber, functionalization, HMF, LA, hydrogenation

## Abstract

Carbon nanofibers (CNFs) have been functionalized by introducing O, N, and P containing groups in order to investigate the effect of support functionalization in Ru catalysed hydroxymethyl furfural (HMF) and levulinic acid (LA) hydrogenation. In the case of HMF, despite the fact that no effect on selectivity was observed (all the catalysts produced selectively gamma-valerolactone (GVL)), the functionalization strongly affected the activity of the reaction. O-containing and N-containing supports presented a higher activity compared to the bare support. On the contrary, in HMF hydrogenation, functionalization of the support did not have a beneficial effect on the activity of a Ru-catalysed reaction with respect to bare support and only CNFs-O behaved similarly to bare CNFs. In fact, when CNFs-N or CNFs-P were used as the supports, a lower activity was observed, as well as a change in selectivity in which the production of ethers (from the reaction with the solvent) greatly increased.

## 1. Introduction

The discovery of carbon nanofibers (CNFs) dates back to 1889, when “hair-like” carbon filaments were reported to grow on metal crucible walls through decomposition of an organic gas at a high temperature [1]. It is only in the last few decades, however, that CNFs have attracted considerable attention. Iijima et al., in particular, were the first to characterize in detail such nanostructures, reporting high thermal and electrical conductivity, high mechanical strength, and good chemical resistance [2]. Currently, CNFs are widely used in gas sensors, as polymer additive, in electronic components, as drug delivery systems and as heterogeneous catalysts [3,4,5,6,7].

The incredibly easy tunability of some physiochemical properties, such as a surface area, porosity, and surface chemistry, makes these materials extremely versatile from a catalytic point of view [5,6]. The possibility of supporting transition metal nanoparticles on the inner walls allows to use them as nanoreactors, while the wide range of functionalization that can be introduced on the external surface, allows them to improve their dispersion in a broad range of solvents as well as to easily anchor both metal nanoparticles and organic molecules [8,9,10]. Moreover, depending on the type of functionalization introduced, the surface acidity/basicity of the support can be modified in order to increase the selectivity towards specific products [11]. For example, the presence of N-containing functionalization on CNFs proved to enhance the activity and stability of Pd and AuPd nanoparticles in the liquid phase oxidation of benzyl alcohol through an increase in metal-support interaction [12,13]. In addition, the nature of the functionalization can affect the reaction pathway as well. Pd nanoparticles deposited on pyridine-containing CNFs, in fact, showed higher activity in the same reaction compared to pyrrole/pyridine-containing ones due to a higher surface basicity that facilitated the H-abstraction step [14]. O-containing functionalities proved to be critical in the Pd-catalyzed cinnamaldehyde hydrogenation reaction. The presence of oxygen functionalities impeded the adsorption of the benzene ring on the CNFs surface, which increased the selectivity towards the hydrogenation of the carbonyl group [15,16,17]. In addition, in a recent report, we showed an efficient procedure for the introduction of C-O-P functionalities on CNFs and we demonstrated their activity in the metal-free fructose dehydration to HMF [18].

**Figure 1:**
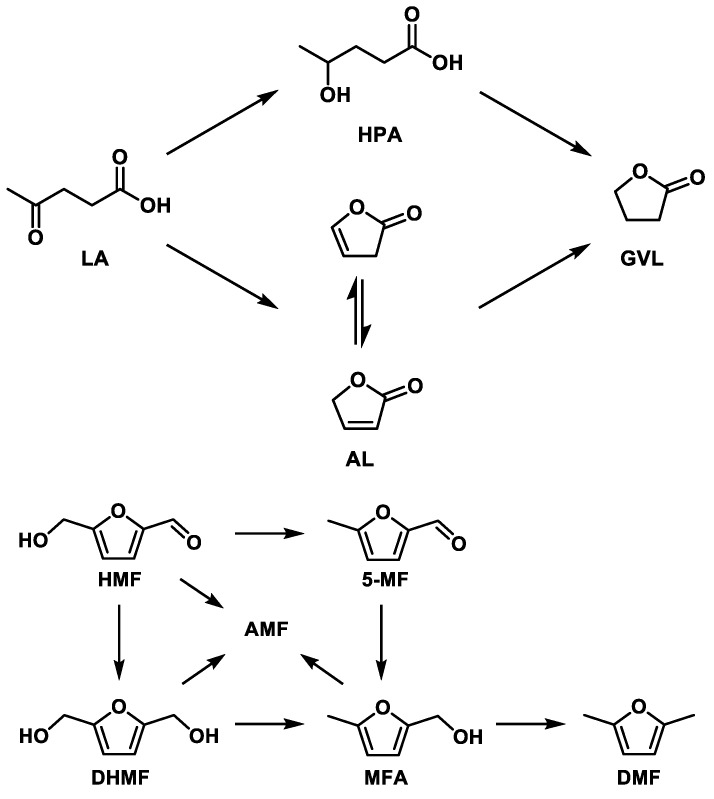
Typical Ru catalyzed reaction mechanism for (top) the conversion of levulinic acid (LA) to gamma valerolactone (GVL) and (bottom) the conversion of 5-hydroxymethylfurfural (HMF) in an alcoholic solvent.

In this study, we focussed our attention on the importance of CNFs functionalization in the hydrogenation of two important biomass derived chemical platforms, namely levulinic acid (LA) and 5-hydroxymethylfurfural (HMF). Both molecules have attracted more attention in the past several years as potential renewable feedstock directly derived from cellulose for the production of fuels and fine chemicals [19,20,21,22,23,24,25,26,27]. The hydrogenation of levulinic acid leads primarily to gamma-valerolactone (GVL) through either formation of 4-hydroxypentanoic acid (HPA) or angelica lactone (AL) as intermediates (Figure 1-top) [28,29]. GVL can be used as is, since it is an additive for biofuels, or as solvent, while it can further be converted into high added-value chemical intermediates for the production of biopolymers or into more refined liquid fuels [30,31,32,33]. 

The hydrogenation of HMF, on the other hand, is a much more complicated reaction, and several products can be formed (Figure 1). 2,5-dimethylfuran (DMF), for example, is a well-known potential biofuel with a high research octane number and energy density [34,35,36], while 2,5-dihydroxymethylfuran (DHMF) can be used in the polymer industry to produce polyethers based polyurethane foams, as a solvent or as a precursor to the production of other high-value chemicals [37,38]. Moreover, the products of etherification of HMF with alcoholic solvents (AMF, alkoxymethyl furfurals), have recently attracted attention due to their potential use as fuel or fuel additives [39,40,41]. The use of an alcoholic solvent in this latter reaction is recommended due to the instability of HMF in water [42].

Several studies have been conducted on Ru nanoparticles supported on carbonaceous materials for the cited reactions [28,29,43,44,45,46,47,48]. Ru, in fact, is considered one of the most effective catalyst due to the high activity displayed and the good selectivity toward the desired products. This is mainly due to the strong ability of Ru to interact with O-containing species and promote the hydrodeoxygenation pathway [49,50].

In a recent publication, in particular, we reported the use of Ru nanoparticles supported on activated carbon and carbon nanofibers for the selective production of DMF, DHMF, and AMF [51]. We demonstrated that the selectivity of the reaction could be tuned simply by using a different support. In particular, Ru supported on CNFs displayed high conversions of HMF into DHMF and AMF with only a small formation of products of hydrogenolysis (DMF). In this work, CNFs have been treated in order to introduce different types of functionalization known as N-functionalization (CNFs-N), O-functionalization (CNFs-O), and P-functionalization (CNFs-P), with the aim to study how the nature of different functionalities affects the activity and selectivity of liquid phase hydrogenation reactions. For this purpose, the catalysts were first characterized with High-Resolution Transmission Electron Microscopy (HRTEM) and X-ray Photoelectron Spectroscopy (XPS), and then tested in the liquid phase hydrogenation of LA and HMF.

## 2. Results and Discussion

### 2.1. Catalysts Characterization

Commercial hollow fishbone pyrolytically stripped fibers (CNFs-PS) were used (simply referred as CNFs). The fibers possess a thin chemically vapor deposited (CVD) layer of carbon over a graphitic fishbone core. The pyrolytical stripping step allows us to remove any polyaromatic hydrocarbons from the fiber surface, which leaves a thin surface layer of amorphous carbon. The calculated fibers’ average diameter was 80 ± 30 nm and had a specific surface area of ca. 50 m^2^ g^−1^. This support was then treated as reported in the Materials and Methods section in order to introduce different functionalization known as nitrogen (CNFs-N), oxygen (CNFs-O), and phosphorous (CNFs-P) containing groups.

The three different functionalized supports were fully characterized by X-ray Photoelectron Spectroscopy (XPS) in order to obtain information about the presence and the abundance of the functionalities on the support surface. The analyses were conducted on the 1s orbital of all the three heteroatoms, and peak assignments were made according to previous results [18,52]. Table 1, in particular, shows the atomic ratio between C and the different functionalities introduced, while Table 2 shows the nature of the different functional groups detected. It should be noted that oxygen is always present in all the samples, either as individual functionality or conjugated with other heteroatoms. This is expected since all the supports underwent an oxidative pre-treatment. 

The pristine CNFs sample showed only the presence of small amounts of surface O-functionalities (2.9%), while no N or P functional groups were detected (Table 1). N-functionalized CNFs, on the other hand, showed the presence of both N and O functionalities, with a similar relative surface abundance of these two heteroatoms (3.8% for both N and O, Table 1). N-containing species were mostly composed of pyridine and pyrrole/pyridone groups (49.3% and 44.1%, respectively, at BE of 398.3 and 400.2 eV). The abundance of pyridone groups was validated by the high concentration of C=O groups detected in the O 1s spectrum (43.4%, at a BE of 531.5). Quaternary N^+^ and pyridine oxide N^+^-O^−^ groups were present in a small amount; the results show 3.5% and 3.1% of the total nitrogen-containing species at BE of 401.2 and 404.6 eV respectively. Apart from C=O groups, a high amount of C-O and C-O-C groups were also detected in the CNFs-N sample (53.2% at a BE of 533.6 eV), while the remaining oxygen was present as adsorbed H_2_O (3.4% at a BE of 536.2 eV). In the CNFs-O sample, oxygen represented the 14.8% of the surface atoms detected (Table 1). Most of the O-functionalities were present as a carbonyl group (C=O, BE of 531.3 eV, which represents the 53.2% of the total oxygenated species), while the remaining functionalities were composed of single C-O bonds (alcoholic and ether groups, BE of 533.1 eV, representing the 42.4% of the total oxygenated species). The remaining 4.4% of oxygen detected in the sample was present as adsorbed H_2_O (BE 534.5 eV). Lastly, the CNFs-P support showed only a small presence of P functionalities (0.3%) as phosphate groups (C-O-P, at a BE of 133.7 eV). As for the other supports, the O 1s spectrum showed a high amount of both carbonyl and alcoholic/ether groups (50.1% and 44.5% at 531.6 and 533.3 eV, respectively). In this case, however, the two peaks overlap with the P=O, P-O, and P-O-C groups characteristic of phosphorylated carbonaceous materials. The remaining 5.4% of oxygen detected at 535.6 eV is present as adsorbed H_2_O.

The XPS analysis of the C 1s region is reported in Table 2. Four different components could be identified in a BE range of 284–292 eV and were assigned according to previous results [18]. In particular, the peak at ca. 284 eV corresponded to sp^2^ graphitic carbon, the peak at ca. 285–286 eV to sp^3^ carbon and C-P bonds, the peak at ca. 288 eV to carbon-oxygen bonds, and the peak at ca. 291 eV to aromatic rings. The relative abundance of the four species did not change significantly, which indicates that the nature of the functionalization affects only in minimal part the CNFs structure.

**Figure 2:**
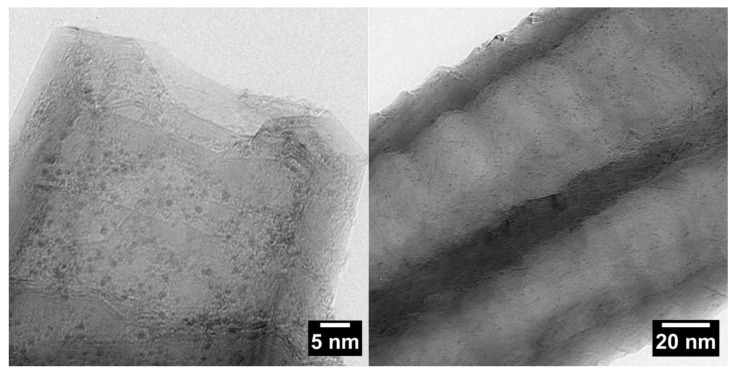
Representative TEM micrograph of the Ru/CNFs catalyst.

After the deposition of Ru by incipient wetness and reduction in H_2_, TEM analyses performed on the four catalysts showed a similar average particle size for all the samples in the range of 1 to 2 nm. A good particle dispersion was observed in all cases, which is shown for Ru/CNFs in Figure 2.

### 2.2. Catalytic Results—LA Hydrogenation

**Figure 3:**
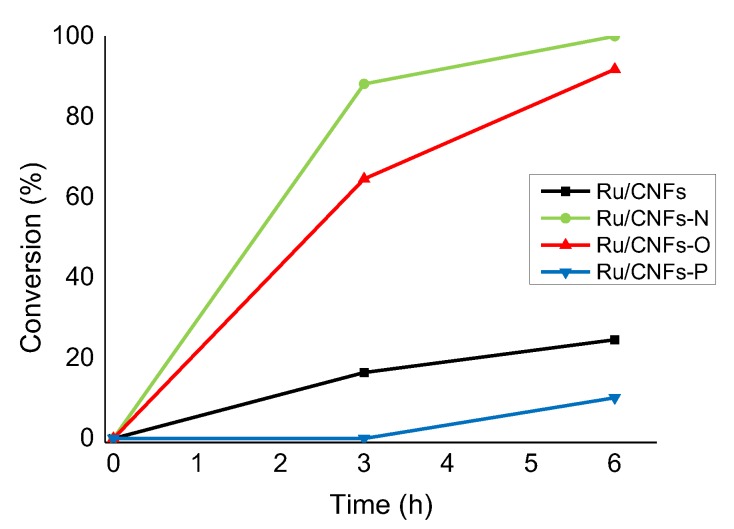
Change in activity in the LA hydrogenation reaction on the different catalysts tested. All the substrate selectively converted to GVL (selectivity > 99%). Reaction conditions: LA, 0.3 M, substrate/metal = 1000 mol/mol, solvent, 15 mL H_2_O, 100 °C, 7 bar H_2_.

The influence of support functionalization on the catalytic performance was first investigated in the hydrogenation reaction of LA. The reaction conditions chosen were 100 °C and 7 bar of H_2_, using water as a solvent (LA concentration of 0.3 mol L^−1^; Ru:LA molar ratio of 1:1000). Before the reaction, the catalyst was activated in autoclave at 200 °C and 5 bar of H_2_ for 1 h, as described in the Materials and Methods section. Previous reports showed the importance of the acid/base properties in tuning the activity of Ru-catalysed hydrogenation of levulinic acid, which demonstrates that, in the presence of strong acidic sites, the conversion increased even though part of the produced GVL was further converted into pentanoic acid [47]. In our case, despite the presence of functionalities of a different nature, the selectivity of the reaction was not affected, with GVL being the only product detected. Although, based on our results, it is impossible to predict the reaction mechanism, based on previous reports, we assumed GVL was formed by the first reduction to HPA followed by a final dehydration step [33,53]. The impact on the catalytic activity, however, was significant and the results are presented in Figure 3. The presence of N and O functionalities greatly enhanced the activity, which increased the LA conversion after 3 h from 16% with CNFs as supported to 65% and 88% with CNFs-O and CNFs-N, respectively. Since the metal dispersion is comparable in all the catalysts, we tentatively ascribed this effect to the increased hydrophilicity of CNFs-O and CNFs-N with respect to pristine CNFs. The low activity of Ru nanoparticles supported on bare CNFs can be ascribed to the low dispersion of the catalyst in water due to the high hydrophobicity of the un-functionalized support [54]. However, considering the strong deactivation observed in the case of Ru on CNFs-P, we excluded this explanation and ascribed the positive effect of CNFs-O and CNFs-N to the presence of O and N functionalities. The presence of weak acid sites (carbonyl, alcoholic, and ether groups, Table 2) on the CNFs-O surface could explain the boost in activity when Ru/CNFs-O was used as a catalyst. The increase in activity in the presence of weak basic N-functionalities, on the other hand, could be attributed to the strong metal-support interaction between the N-containing groups and the anchored metallic Ru nanoparticles. In fact, similar results were obtained with N-doped CNFs in the Pd-catalyzed benzyl alcohol oxidation reaction. The enhanced activity was attributed to strong metal-N interactions that prevented agglomeration and sintering phenomena of the metal nanoparticles as well as metal leaching [12,13,14]. With the introduction of P-containing functionalities, we observed only 10% of LA conversion after 6 h of reactions. This is in contrast with recent results, where an increase in P content resulted in an increase in the conversion rate of the fructose dehydration reaction with metal-free P-functionalized CNFs [18].

### 2.3. Catalytic Results—HMF Hydrogenation

**Figure 4:**
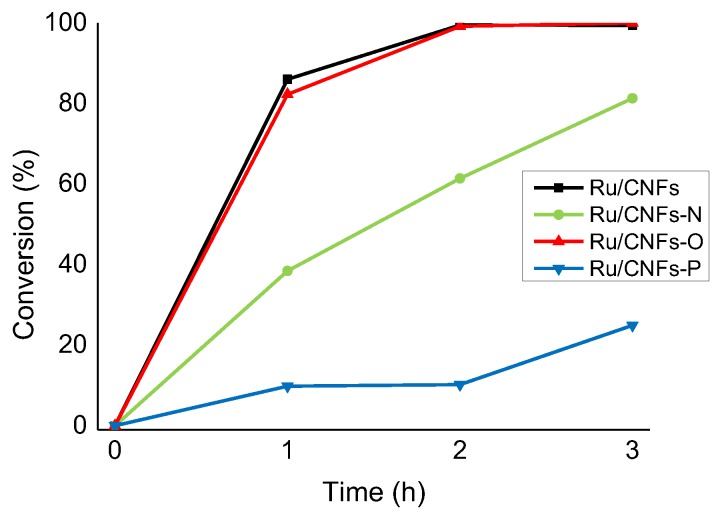
Change in activity in the HMF hydrogenation reaction on the different catalysts tested. Reaction conditions: HMF, 0.08 M, substrate/metal = 100 mol/mol, solvent, 15 mL 2-butanol, 150 °C, and 20 bar H_2_.

**Figure 5:**
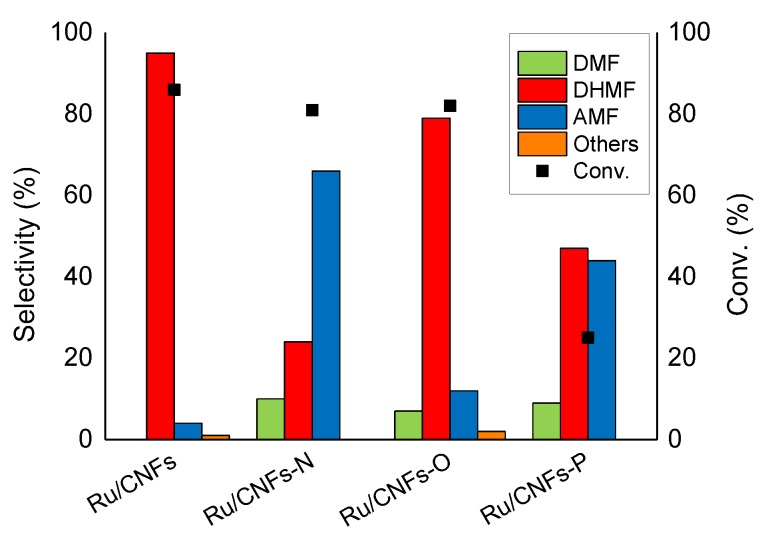
Change in selectivity in the HMF hydrogenation reaction on the different catalysts tested. Reaction conditions: HMF, 0.08 M, substrate/metal = 100 mol/mol, solvent, 15 mL 2-butanol, 150 °C, 20 bar H_2_, reaction time: Ru/CNFs and Ru/CNFs-O 1 h, Ru/CNFs-N and Ru/CNFs-P 3 h.

The hydrogenation of HMF was performed in 2-butanol as solvent at 150 °C and 20 bar of H_2_ (HMF concentration of 0.08 mol L^−1^, Ru:HMF molar ratio of 1:100), with each catalyst being activated in an autoclave at 200 °C and 5 bar of H_2_ for 1 h before reaction. 2-butanol was used since it is one of the most used extraction solvents in the fructose dehydration to HMF [35,37,55,56]. A graph comparing the activity of the different catalysts is reported in Figure 4. The presence of an organic medium in the HMF hydrogenation, annul the negative hydrophobicity effect observed in the LA hydrogenation with Ru/CNFs. In this case, in fact, Ru nanoparticles supported on bare CNFs displayed the highest activity among the catalysts studied (86% of conversion after 1 h). The presence of weak acid sites (CNFs-O) in this case appeared to not have an effect on the catalyst behavior. Ru on CNFs and CNFs-O behaved similarly in terms of activity and selectivity (82% of conversion after 1 h) (Figure 4 and Figure 5). Apart from LA hydrogenation, the N-functionalized CNFs catalyst showed a detrimental effect in activity (40% of conversion after 1 h) (Figure 4). However, most importantly, they catalyzed the formation of ethers more efficiently by reacting DHMF and methylfurfuryl alcohol (MFA) with the solvent (Figure 5).

Lastly, in this case, the Ru/CNFs-P catalysts showed the lowest activity among the catalysts tested (25% of conversion after 3 h). Despite the low content of P-functionalities (Table 1), it appears that the P-groups have a strong negative effect on the catalyst activity in contrast with what was reported for dehydration reactions [18]. This presented a selectivity similar to the one showed by CNFs-N.

Considering more in details the selectivity of this complex reaction, a previous study reported the hydrogenation of HMF in the presence of a CNFs-based catalyst producing a series of products derived from several consecutive and parallel reactions (Figure 1-bottom) [51]. The selectivity in Figure 5 were compared at iso-conversion (80%, except with Ru/CNFs-P that was compared at ca. 30% of conversion). The main products observed were DHMF, DMF, and AMF, with the latter being a mixture of ethers derived from the reaction between DHMF and methylfurfuryl alcohol (MFA) with the solvent (See Supplementary Materials).

**Figure 6:**
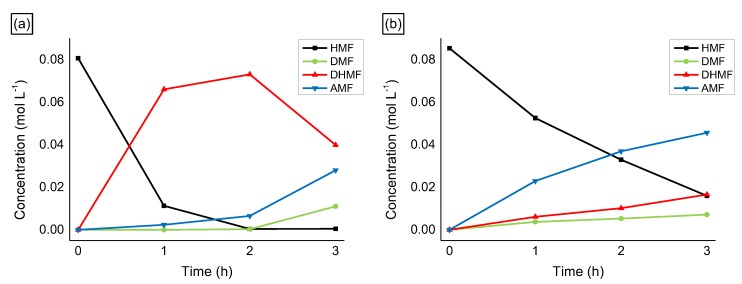
HMF hydrogenation reaction profile on (**a**) Ru/CNFs and on (**b**) Ru/CNFs-N. Reaction conditions: HMF, 0.08 M, substrate/metal = 100 mol/mol, solvent, 15 mL 2-butanol, 150 °C, 20 bar H_2_.

Very high DHMF selectivity was obtained on Ru nanoparticles supported on un-functionalized CNFs (up to 95%), with only a small amount of AMF produced. The low functionalization of CNFs probably inhibits the etherification process that usually requires acid/basic sites to happen [45,57]. In this case, the hydrogenation reactions prevail on the support catalyzed reaction. On the other hand, when N-functionalities were introduced on the CNFs, the selectivity drastically changed towards a high production of AMF (66%) with only 24% of the products being DHMF. It is not the first time that ethers formation is reported on N-doped carbon-based catalysts. Products of etherification between furfuryl alcohol and the alcohol solvent were observed in the hydrogenation transfer reaction of the furfural to furfuryl alcohol [58]. It is interesting to notice that, with Ru/CNFs-N, small amounts of DMF were produced as well (ca. 10% of selectivity). Comparing the reaction profile of the reactions catalyzed by Ru/CNFs and Ru/CNFs-N (Figure 6a,b), it is possible to observe that DMF is only produced at a later stage when bare CNFs are used as a support. In particular, once all the substrate is consumed, the DHMF produced starts to convert into AMF and DMF (35% and 14% of selectivity after 3 h of a reaction). On the other hand, when N-functionalized fibers were used, both AMF and DMF were produced since the early stage of the reaction (70% and 11% of selectivity after 1 h of a reaction).

**Figure 7:**
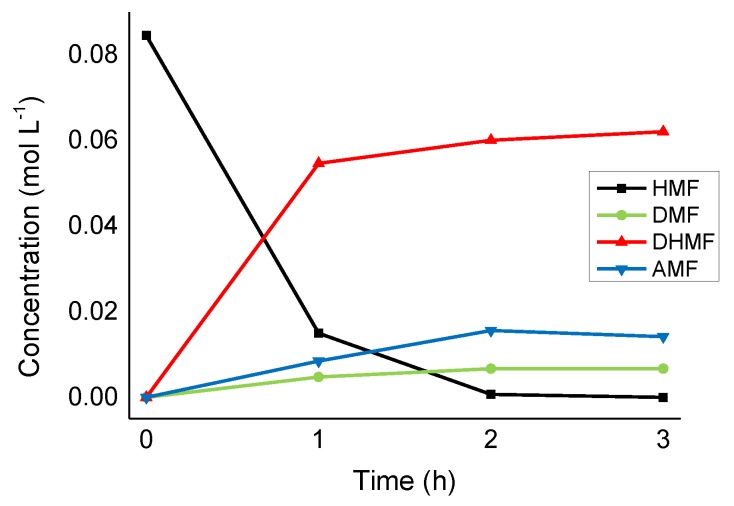
HMF hydrogenation reaction profile on Ru/CNFs-O. Reaction conditions: HMF, 0.08 M, substrate/metal = 100 mol/mol, solvent, 15 mL 2-butanol, 150 °C, 20 bar H_2_.

Similarly to Ru/CNFs, Ru nanoparticles supported on O-functionalized fibers displayed high selectivity toward the production of DHMF (79% of selectivity after 1 h of reaction). In this case, however, the DHMF produced did not show a tendency to further convert into AMF and DMF even once all the initial HMF was fully converted (Figure 7). The selectivity towards DHMF, in fact, did not vary significantly at extended reaction times (from 73% to 75% at 2 and 3 h of reaction, respectively), which suggests that DHMF is a stable product in the presence of the Ru/CNFs-O catalyst. 

**Figure 8:**
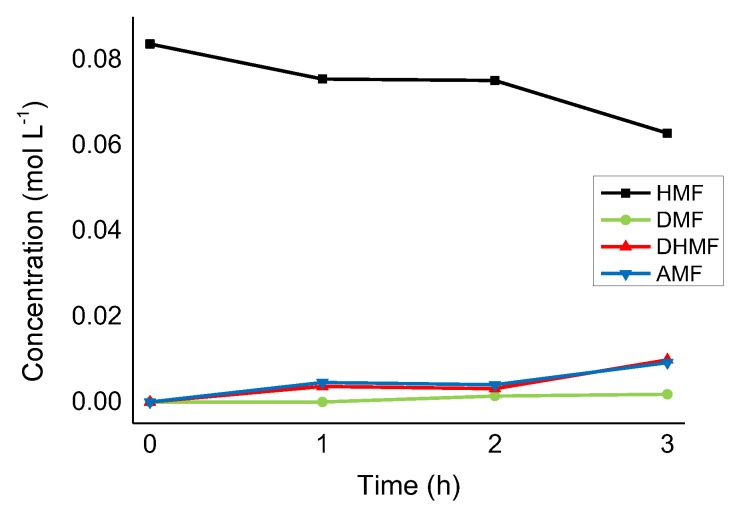
HMF hydrogenation reaction profile on Ru/CNFs-P. Reaction conditions: HMF, 0.08 M, substrate/metal = 100 mol/mol, solvent, 15 mL 2-butanol, 150 °C, 20 bar H_2_.

Lastly, the Ru/CNFs-P catalyst showed not only a low catalytic activity (Figure 5) but also poor product selectivity (Figure 8), with both DHMF and AMF being produced in a similar amount (47% and 44% of selectivity after 3 h of the reaction).

## 3. Materials and Methods 

### 3.1. Support Functionalization

Commercial CNFs were used in this study as is (CNFs PR24-PS, Applied Science Inc., Cedarville, OH, USA) or opportunely functionalized.

CNFs-O: The oxygen-containing nanocarbons were obtained by treating the pristine support with HNO_3_ according to the following procedure: a solution of CNFs in concentrated HNO_3_ (20 g of CNF per liter of HNO_3_) was kept at 373 K for 2 h under continuous stirring. It was then rinsed with distilled water, and dried at 343 K for several h.

CNFs-N: N-containing CNFs were obtained from the pre-oxidized CNFs (CNFs-O) by thermal treatment (10 g for each batch) with NH_3_ at 873 K for 4 h.

CNFs-P: For the P-functionalization 2 grams of the CNF sample were suspended in 250 mL of a HNO_3_-H_3_PO_4_ 1:1 vol/vol mixture, stirred, and heated at 150 °C (2 h). 

The functionalized carbon samples were then filtered, thoroughly washed with distilled water until reaching neutrality, and dried at 100 °C overnight.

### 3.2. Catalyst Preparation

The catalysts were prepared by incipient wetness impregnation method using an aqueous solution of RuCl_3_∙xH_2_O (Sigma-Aldrich, Haverhill, MA, USA, 99.98%). The appropriate amount of Ru precursor was dissolved in deionised water in order to obtain a final volume that corresponded to the specific pore volume of the support. Each support pore volume was measured experimentally by consecutive addition of 100 µL of deionised water to a 1 g sample of CNFs until complete impregnation was reached (Table 3). The solution was added in the desired amount of support placed into a glass vial and manually stirred with a glass rod until the sample homogeneity was reached. The black slurry was dried in an oven at 80 °C for 16 h. Before use, the catalyst was activated in the autoclave at 200 °C and 5 bar of H_2_ for 1 h.

### 3.3. LA Hydrogenation Reactions

The LA hydrogenation reactions were carried out in a 100 mL stainless steel autoclave. In a typical experiment, 15 mL of a 0.3 mol L^−1^ aqueous solution of LA (Sigma-Aldrich, 99%) and an appropriate amount of activated catalyst (LA:Ru molar ratio of 1:1000) were placed into the autoclave along with a magnetic stirrer. The system was flushed several times with N_2_ first in order to remove any residual oxygen in the atmosphere and then pressurised with 7 bar of H_2_. The reaction was then heated at the desired temperature (100 °C) and the solution stirred at a constant stirring of 1250 rpm. Samplings were carried out by stopping the stirring and quenching of the reaction in an ice bath. A sample of solution was withdrawn (ca. 300 µL), centrifuged in order to separate the catalyst, and 100 µL of the supernatant solution diluted into 5 mL of a 1 wt% solution of H_3_PO_4_. The solution was injected as is into an HPLC system equipped with an Alltech OA-10308 column (Hichrom, Lutterworth, UK, 300 mm × 7.8 mm), UV, and a refractive index detector. A 1 wt% solution of H_3_PO_4_ was used as eluent. Peak identification was carried out by comparison with the original samples.

### 3.4. HMF Hydrogenation Reactions

The HMF hydrogenation reactions were carried out in a 100 mL stainless steel autoclave equipped with a mechanical stirrer and a thermocouple. In a typical experiment, 0.1550 g of HMF (Sigma-Aldrich, 99%) were dissolved in 15 mL of 2-butanol (Sigma-Aldrich, 99%). The solution was placed into a glass inlet with the appropriate amount of activated catalyst (Ru:HMF molar ratio of 1:100). The system was then sealed and purged with N_2_ first and then H_2_, which pressurized the reactor to the desired pressure (20 bar). The autoclave was heated up to 150 °C and the solution was stirred at 1000 rpm. Samplings were carried out by stopping the stirring and quenching of the reaction of the samples in an ice bath. A sample of the reaction solution (ca. 500 µL) was withdrawn and centrifuged in order to separate the catalyst from the solution. The filtrate was diluted with a solution of an external standard (dodecanol, Sigma-Aldrich, >98%) for GC measurement. Product analysis was carried out with a GC-MS (Thermo Scientific, Waltham, MA, USA, ISQ QD equipped with an Agilent VF-5ms column) and the resulting fragmentation peaks were compared with standards present in the software database. Product quantification was carried out through a GC-FID equipped with a non-polar column (Thermo Scientific, TRACE 1300 equipped with an Agilent HP-5 column).

### 3.5. Catalysts Characterization

X-ray photoelectron spectroscopy (XPS) was performed on a Thermo Scientific K-alpha+ spectrometer. Samples were analyzed using a monochromatic Al x-ray source operating at 72 W (6 mA × 12 kV), with the signal averaged over an oval-shaped area of approximately 600 × 400 microns. Data was recorded at pass energies of 150 eV for survey scans and 40 eV for a high resolution scan with a 1 eV and 0.1 eV step size, respectively. Charge neutralization of the sample was achieved using a combination of both low energy electrons and argon ions (less than 1 eV), which gave a C(1s) binding energy of 284.8 eV.

All data were analyzed using CasaXPS (Microsoft Corporation, Redmond, WA, USA) (v2.3.17 PR1.1) using Scofield sensitivity factors and an energy exponent of −0.6. 

Scanning transmission electron microscopy (STEM) data were collected on the Ru catalysts by using a Hitachi H3300 STEM operated at 200 kV in the Z-contrast mode in which the brightness depended on the thickness and, approximately, the square of the atomic number. Particle sizes were determined by using ImageJ software to process the STEM images.

## 4. Conclusions

Carbon nanofibers have been functionalized introducing oxygen, nitrogen, and phosphorous-containing groups. These materials have been compared as supports for Ru nanoparticles to be used in the hydrogenation of HMF and LA. Similar metal dispersion were obtained in all cases but the activity of the catalysts differed greatly. CNFs-N appeared as the best support in the Ru-catalyzed LA hydrogenation in water, superior to pristine CNFs. The higher hydrophilicity of the support did not appear as the only reason for the high activity but, likely, the strong interaction of N-groups with Ru particles plays a role. Ru on pristine CNFs presented comparable activity/selectivity as Ru on CNF-O in HMF hydrogenation in 2-butanol. On the contrary, Ru/CNF-N and Ru/CNF-P showed a lower activity but also a change in selectivity. In fact, these latter two catalysts enhanced the formation of ethers due to the reaction between DHMF and methylfurfuryl alcohol (MFA) with the solvent.

## Figures and Tables

**Table 1:** C:N:O:P Atomic ratio calculated by XPS analysis of the various supports.

Sample	Atomic Ratio % C:N:O:P
CNFs	97.1:0:2.9:0
CNFs-N	92.0:3.8:3.8:0
CNFs-O	85.2:0:14.8:0
CNFs-P	87.3:0:12.4:0.3

**Table 2:** XPS analysis of the different supports used.

Sample		N 1s				O 1s			P 1s	C 1s			
		Pyridine	Pyrrole/PyRidone	Quaternary N^+^	Pyridin Oxide	C=O, P=O, P-O	C-O, C-O-C, P-O-C	H_2_O	C-O-P	sp^2^	sp^3^, C-P	C-O, C=O	C=C
CNFs-N	BE eV	398.3	400.2	401.2	404.6	531.5	533.6	536.2	-	284.7	286.2	288.1	291.7
	Atom %	49.3	44.1	3.5	3.1	43.4	53.2	3.4		73.1	13.4	8.8	4.8
CNFs-O	BE eV	-	-	-	-	531.3	533.1	534.5	-	284.6	285.1	288.4	291.5
	Atom %					53.2	42.4	4.4		76.8	13.2	6.8	3.2
CNFs-P	BE eV	-	-	-	-	531.6	533.3	535.6	133.7	284.5	285	288.5	291.1
	Atom %					50.1	44.5	5.4	100	77.4	14.4	5.7	2.5

**Table 3:** Specific support pore volume.

Support	Pore Volume (mL g^−1^)
CNFs	11.0
CNFs-N	7.2
CNFs-O	5.1
CNFs-P	8.3

## Supplementary Materials

The following are available online. Figure S1: Products of etherification of dihydroxymethylfuran (DHMF) and furfuryl alcohol (MFA) with the solvent 2-butanol. Figure S2: GC-MS analysis of the AMF detected in the reaction mixture.
