# Supplementary material for: CNF-Functionalization as Versatile Tool for Tuning Activity in Cellulose-Derived Product Hydrogenation
Source: Molecules. 2019 Jan 16;24(2):316. doi: 10.3390/molecules24020316 (PMC6359071; doi:10.3390/molecules24020316)

Supplementary Information

CNF-functionalisation as versatile tool for tuning activity in cellulose-derived product hydrogenation

Andrea Jouve[1], Stefano Cattaneo[1], Sofia Capelli[1], Marta Stucchi[1], Claudio Evangelisti[2], Alberto Villa[1] and Laura Prati[1,*]

[1] Dipartimento di Chimica, Università degli Studi di Milano, via Golgi 19, I-20133 Milano, Italy;
[2] National Council of the Research, CNR-ISTM, Via G. Fantoli 16/15, 20138 Milan, Italy;
* Correspondence: laura.prati@unimi.it; Tel.: +39-0250-314-357

Figure S1. Products of etherification of dihydroxymethylfuran (DHMF) and furfuryl alcohol (MFA) with the solvent 2-butanol.

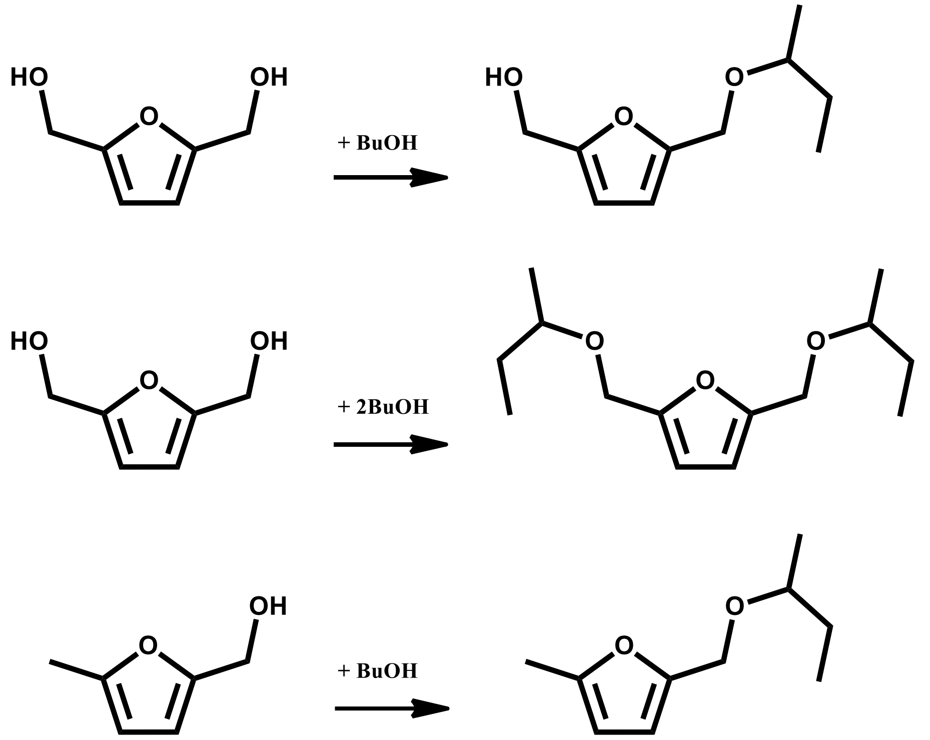

Figure S2. GC-MS analysis of the AMF detected in the reaction mixture.

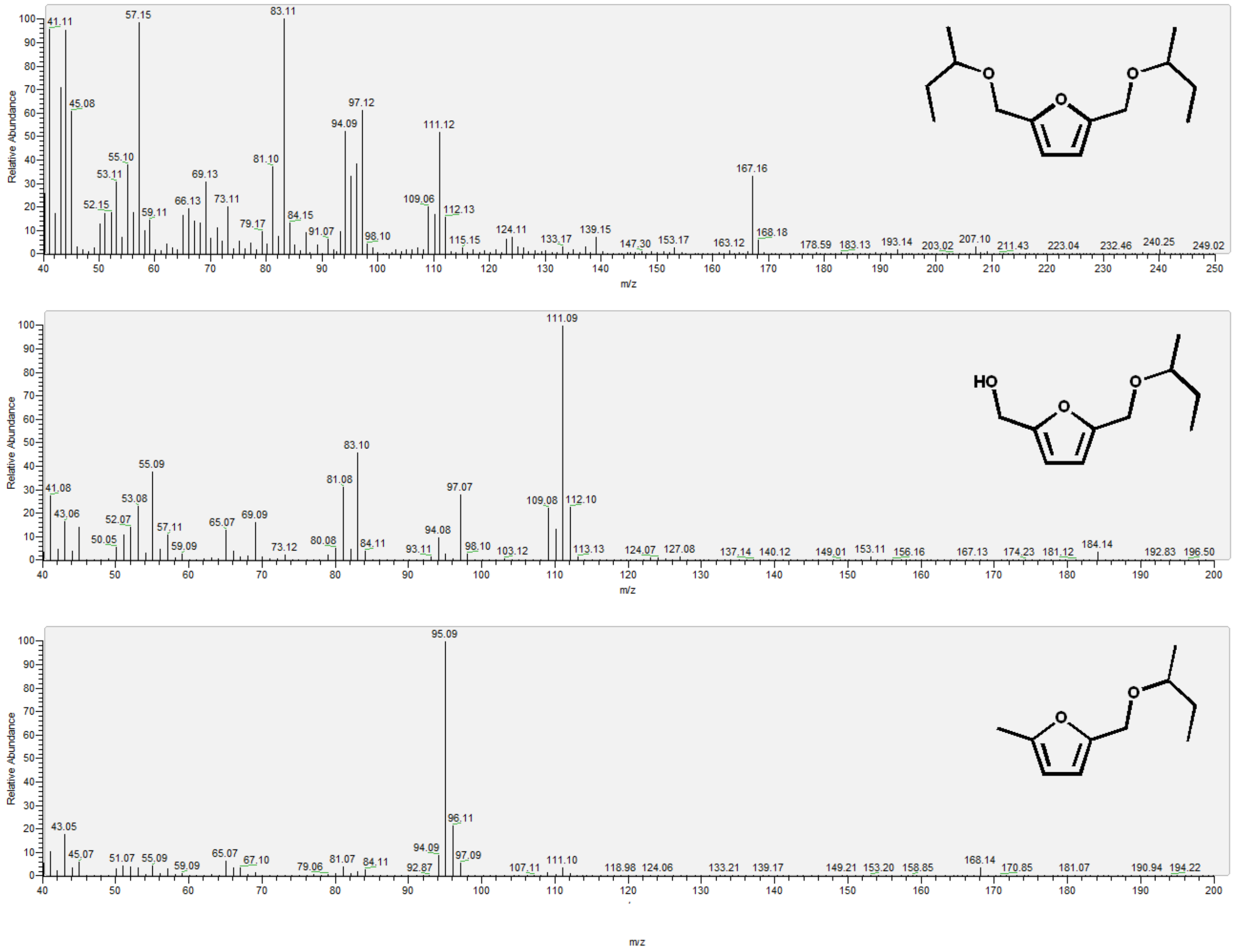

Supplement: Supplementary file 1 [file molecules-24-00316-s001.pdf]
